# A retrospective study on the clinicopathological and molecular features of 22 cases of natural killer/T-cell lymphoma in children and adolescents

**DOI:** 10.1038/s41598-022-11247-z

**Published:** 2022-05-03

**Authors:** Guan‑Nan Wang, Wu‑Gan Zhao, Xu-Dong Zhang, Xiang-Yu Jian, Chong-Li Zhang, Ming-Zhi Zhang, Wen‑Cai Li

**Affiliations:** 1 Department of Pathology, The First Affiliated Hospital of Zhengzhou University, 1 Jianshe Road, Zhengzhou, 450052 Henan People’s Republic of China; 2 Department of Oncology, The First Affiliated Hospital of Zhengzhou University, 1 Jianshe Road, Zhengzhou, 450052 Henan People’s Republic of China

**Keywords:** Cancer, Computational biology and bioinformatics, Genetics, Diseases, Oncology

## Abstract

Natural killer/T-cell lymphoma (NKTCL) in children and adolescents is a rare type of T/NK cell neoplasms. The aim of the present study was to analyze the clinicopathological and genetic features of this rare entity of lymphoma. We evaluated the clinical, histopathological and molecular features of 22 young people with NKTCL, including 15 males and 7 females, with a median age of 15 years. The results revealed that the nasal site was the most involved region while non-nasal sites were observed in 27.3% out of all cases. The tumor cells were composed of small‑sized to large cells and 19 (86.4%) cases exhibited coagulative necrosis. The neoplastic cells in all patients were positive for CD3 and the cytotoxic markers. Nineteen (86.4%) cases were positive for CD56. Reduced expression of CD5 was observed in all available cases. CD30 was heterogeneously expressed in 15 (75.0%) cases. All 22 patients were EBV positive. Seven (36.8%) out of all the 19 patients during the follow-up died of the disease, and the median follow‑up period was 44 months. Moreover, patients treated with radiotherapy/chemotherapy showed significantly inferior OS compared with the untreated patients. High mutation frequencies were detected including KMT2C (5/5), MST1 (5/5), HLA-A (3/5) and BCL11A (3/5), which involved in modifications, tumor suppression and immune surveillance. These results suggest that NKTCL in children and adolescents exhibits histopathological and immunohistochemical features similar to the cases in adults. Active treatment is necessary after the diagnosis of NKTCL is confirmed. Furthermore, genetic analyse may provide a deep understanding of this rare disease.

## Introduction

Natural killer/T-cell lymphoma (NKTCL) is a major type of natural killer and T cells neoplasm, which is considered to be an aggressive disease with distinct clinical and histopathological features, characterized by Epstein–Barr virus (EBV) infection. The incidence of this disease is higher in Asia, Mexico, and South America than in Western countries^1,2^. In China, NKTCL is the second most common type of lymphoma following diffuse large B-cell lymphoma (DLBCL)^3^. Although NKTCL can occur in children and adolescents, studies with large samples are not available due to the low incidence of NKTCL in this age group^4–7^. Recently, Hang reported 17 NKTCL cases from 2012 to 2014 in which the patients were younger than 17 years of age and having an occupancy of 6% (17/286) of the total sample size during the same period^8^. In a few retrospective analyses of pediatric patients with NKTCL, it has been shown that these patients have some clinical features in common such as male and female distribution were equal or having a slight male predominancy and B-symptoms were often observed with the involvement of non-nasal sites, including the skin, central nervous system, testis, and lungs^5–8^. However, the molecular feature of young patients with NKTCL has not been well-described in these studies and genetic difference between adults and children still remains unknown. In this study, we reviewed the clinicopathologic and genetic features of young patients with NKTCL at our institution in China. The present study may provide a better understanding of the pathophysiological aspects of this rare entity of lymphoma.

## Materials and methods

### Patient selection

Biopsied tissues of patients with NKTCL between May 2012 and March 2019 were identified at the First Affiliated Hospital of Zhengzhou University (Zhengzhou, China). All patients were diagnosed according to the World Health Organization classification (WHO) criteria: the morphological and immunophenotypic characteristics of the tumor cells fulfilled the criteria of NKTCL, and all the cases were EBV positive. The criteria for the enrollment of patients in the present study were based on the following requirements: (1) the maximum age limit for the enrolled patients was eighteen years of age with no minimum age limit. (2) No previous history of chronic EBV-associated illness. (3) Patients with no previous history of treatment for lymphoma. All specimens were routinely processed, embedded in paraffin, sectioned, and stained with hematoxylin and eosin. This study was approved by the First Affiliated Hospital of Zhengzhou University and complies with the Declaration of Helsinki. And the consent obtained from all subjects included in the study and the children’s parents was both informed and written.

### Immunohistochemistry

Immunohistochemistry was performed using 10% formalin-fixed paraffin-embedded tissues that were cut into 4-µm sections, followed by Envision method (a modified avidin‑biotin complex method) on an automated immunostainer (Ventana Medical Systems, Inc., Tucson, AZ, USA). Immunohistochemistry for CD20 (L26, ZSGB-BIO, Beijing, China), CD3 (SP7, Maixin Biotech Co., Ltd., Fuzhou, China), CD5 (SP19, Maixin Biotech Co., Ltd., Fuzhou, China), CD56 (MRQ42, Jiehao Biotech Co., Ltd., Shanghai, China), TIA-1 (2G9A1085, Maixin Biotech Co., Ltd., Fuzhou, China), granzyme B (EP230, ZSGB BIO, Beijing, China), CD30 (UMAB256, ZSGB BIO, Beijing, China), EBNA-2 (M83171, Fitzgerald, USA), and ki-67 (GM001, Gene Tech, Shanghai, China)) was performed (Supplementary Information).

### In situ hybridization (ISH) for EBV

EBV RNA was detected using the Epstein‑Barr Virus Early RNA kit (cat. no. ISH‑5021; OriGene Technologies, Inc.), following the manufacturer's protocol. Briefly, 4–6-µm sections were cut from paraffin‑embedded tissues, deparaffinized with xylene at 37 °C for 10 min, rehydrated, predigested with proteinase K (OriGene Technologies, Inc.), and hybridized with DIG‑labeled RNA probe. Following washing, the reaction was accomplished using anti‑DIG horseradish peroxidase conjugate (OriGene Technologies, Inc.), followed by staining with the 3,3′‑diaminobenzidine substrate at 37 °C for 5 min.

### Sequencing and data processing

Five out of 22 cases with formalin-embedded tissues were eligible for the next-generation sequencing (NGS) test. The criteria for selection as follows: (1) tumor tissues with sufficiently high DNA quality. (2) Formalin-embedded tissues within 5 years. (3) To avoid necrotic areas. Deep sequencing was performed on the Illumina HiSeq4000 platform (Shihe, Nanjing, China). The Genome Analysis Toolkit (GATK)^9^ was used to perform local realignments around indels and base quality reassurance. Single nucleotide variants (SNVs) and short insertions/deletions (indels) were identified using VarScan2 2.3.9^10^ with the minimum variant allele frequency threshold set at 0.01 and the P value threshold for calling variants set at 0.05.

### Statistical analysis

All analyses were performed using SPSS 17.0. Overall survival (OS) rate was defined as the duration between the date of diagnosis and the date of mortality or the last follow-up. Survival rate was analyzed using the Kaplan–Meier method with the log‑rank test.* P* < 0.05 was considered to be statistically significant.

### Ethics approval 

This study was approved by the First Affiliated Hospital of Zhengzhou University. 

### Consent to participate

And the consent obtained from all subjects included in the study and the children’s parents was both informed and written.

### Consent for publication

All the authors agreed to publish our work on this journal.

## Results

### Clinical features

The clinical features of 22 patients with NKTCL are summarized in Table 1. Overall, there were 15 male and 7 female patients (male/female, 2.14:1) with a median age of 15 years and a range of 2–18 years. Among these cases, the nasal site was the most involved region, including the nasal cavity (54.5%, 12/22), nasopharynx (13.6%, 3/22), and maxillary sinus (4.5%, 1/22). Non-nasal sites including skin (9.1%, 2/22), lymph nodes (13.6%, 3/22), and testis (4.5%, 1/22) were observed in 6 (27.3%) out of 22 cases. B-symptoms were frequent (77.3%, 17/22), but BM involvement was detected in only 1 patient (5.0%, 1/20).

**Table 1:** Clinical features of NKTCL in children and adolescents.

Case	Sex/age	Primary site	B-symp	BM	LDH (U/L)	Hb (g/L)	WBC (× 10^9^ /L)	Stage	IPI	Treatment	Duration of follow-up,(mon)	Outcome
1	M/15	Nasopharynx	Yes	No	328	135	10.22	II	2	C + R	67	Alive
2	F/15	Nasal cavity	Yes	No	224	107	10	II	1	C	66	Alive
3	F/18	Nasal cavity	No	No	269	100	5.8	II	2	C + R	58	Dead
4	M/16	Skin of the crus	No	No	280	132	4.2	IV	3	C	57	Alive
5	F/17	Nasal cavity	Yes	No	375	119	4.8	IV	3	C	56	Alive
6	M/13	Nasal cavity	No	No	249	104	8	III	2	No treatment	48	Dead
7	F/17	Nasal cavity	No	No	186	101	4.1	II	1	No treatment	55	Dead
8	M/17	Nasopharynx	Yes	No	230	123	5.6	IV	2	C	51	Alive
9	M/12	Maxillary sinus	Yes	N.A	194	134	17.9	II	1	R	52	Alive
10	F/8	Skin of the arm	Yes	N.A	778	109	3.9	IV	4	No treatment	42	Dead
11	M/18	Nasal cavity	No	No	191	158	11.9	III	2	C	41	Alive
12	M/15	Nasal cavity	Yes	No	321	129	3.9	IV	4	C	N.A	N.A
13	M/18	Nasopharynx	Yes	Yes	706	111	1.5	IV	3	C	40	Alive
14	M/18	Nasal cavity	Yes	No	368	138	7.2	III	3	C + R	44	Dead
15	M/12	Nasal cavity	Yes	No	359	118	6.8	IV	3	C	N.A	N.A
16	M/14	Nasal cavity	Yes	No	344	138	6.4	III	3	C	N.A	N.A
17	M/15	Nasal cavity	Yes	No	201	154	10.2	II	2	C + R	28	Dead
18	M/18	Lymphnodes	Yes	No	1326	131	1.7	IV	3	C	36	Dead
19	M/15	Lymphnodes	Yes	No	550	82	0.8	IV	3	C	36	Alive
20	F/17	Nasal cavity	Yes	No	239	103	5.5	III	2	C + R	17	Alive
21	F/8	Lymphnodes	Yes	No	491	128	3.22	IV	3	C	6	Alive
22	M/2	Testis	Yes	No	235	95	3.26	IV	3	C	2	Alive

*B-symp* B-symptoms at diagnosis, *BM* bone marrow involvement, *N.A.* not available, *LDH* lactic dehydrogenase, *C* chemotherapy, *R* radiotherapy;

The serum lactate dehydrogenase level was elevated in almost half of the patients (59.1%, 13/22). Anemia (< 110 g/L) was observed in 36.4% (8/22) of the patients. Leucopenia (5–12 ×10^9^/L) was observed in 45.5% (10/22) of the cases. Advanced Ann Arbor stage III /IV and high/intermediate IPI were observed in 72.7% (16/22) and 54.5% (12/22) of the patients, respectively.

### Morphological findings

**Figure 1:**
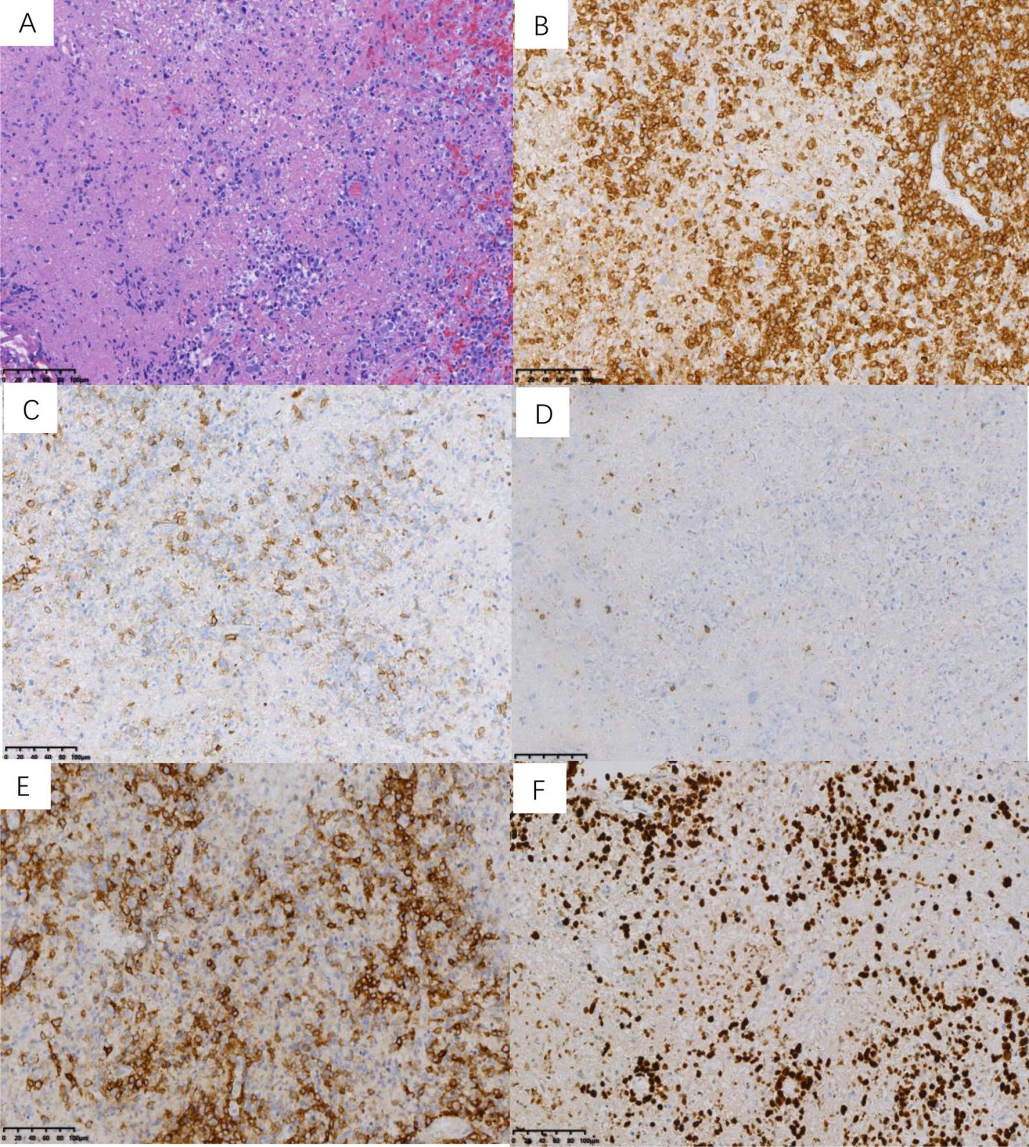
Pathologic features of NKTCL in children and adolescents, lymph nodes (case 21). (**A**) Lymphoid cells were medium-large in size with coagulative necrosis (HE ×200). Neoplastic cells were positive for (**B**) CD3 and (**C**) CD56 (×200). (**D**) CD5 negative was expressed in this case (×200). (**E**) The tumor cells showed focal positivity for CD30 (×200). (**F**) Lymphoid cells were EBV positive by ISH detection, > 100 per high-power field in the hot spot region (×200).

The pathological features of the 22 NKTCL patients are summarized in Table 2. Based on the cell size of tumor cells, NKTCL in the present study could be classified into four histological types: 6 patients with small cell type; 12 patients with medium-size cell type; 2 patients with large cell type; and 2 patients with pleomorphic cell type. Nineteen out of 22 (86.4%) cases exhibited various degrees of coagulative necrosis (Fig. 1A).

**Table 2:** Pathologic features of NKTCL in children and adolescents.

Case	Histological types	Necrosis	CD3	CD5	CD56	Gran-B	TIA-1	CD30	EBNA-2	KI-67 (%)	EBER
1	MC	No	+	Focal +	−	+	+	−	−	60	+
2	SC	Yes	+	Focal +	+	Focal +	+	Focal +	−	80	+
3	SC	Yes	+	Focal +	+	+	+	Diffuse +	−	80	+
4	MC	Yes	+	Focal +	+	+	+	Focal +	−	90	+
5	MC	Yes	+	Focal +	+	+	+	Focal +	−	60	+
6	SC	Yes	+	Focal +	+	+	+	Sporadic +	−	60	+
7	MC	Yes	+	N.A	+	+	+	−	−	80	+
8	SC	Yes	+	N.A	+	+	+	N.A	−	80	+
9	SC	No	+	Focal +	−	−	+	−	−	30	+
10	LC	No	+	−	−	+	+	Sporadic +	−	90	+
11	MC	Yes	+	Focal +	+	+	+	Focal +	−	60	+
12	MC	Yes	+	−	+	+	+	Sporadic +	−	40	+
13	MC	Yes	+	N.A	+	+	+	Focal +	−	70	+
14	MC	Yes	+	N.A	+	+	+	N.A	−	60	+
15	PC	Yes	+	Focal +	+	+	+	Focal +	−	40	+
16	MC	Yes	+	Focal +	+	+	+	−	−	50	+
17	SC	Yes	+	Focal +	+	+	+	Sporadic +	−	60	+
18	PC	Yes	+	Focal +	+	+	+	−	−	70	+
19	MC	Yes	+	Focal +	+	+	+	Diffuse +	−	80	+
20	MC	Yes	+	Focal +	+	+	+	Focal +	−	90	+
21	MC	Yes	+	−	Focal +	+	+	Focal +	−	50	+
22	LC	Yes	+	Focal +	+	+	+	Sporadic +	−	70	+

*SC* small cell type, *MC* medium-sized cell type, *LC* large cell type, *PC* pleomorphic cell type.

### Immunohistochemical analysis and ISH for EBV.

The neoplastic cells in all patients were tested positive for the cytoplasmic CD3 expression (Fig. 1B) and the cytotoxic markers such as granzyme B and TIA-1. The tumor cells were CD56 (Fig. 1C) positive in most cases (86.4%, 19/22), while the other 3 cases were negative for the expression of CD56. Reduced expression of CD5 (Fig. 1D) was observed in all available cases, where 15 (83.3%) cases were focally positive, and 3 cases (16.7%) were negative. CD30 (Fig. 1E) was heterogeneously expressed in 15 out of 20 available cases. In addition, three types of CD30 + expression pattern were observed: sporadic (25.0%, 5 out of 20), focal (40.0%, 8/20), and diffuse (10.0%, 2/20). Proliferative activity of tumor cells was assessed by evaluating Ki-67 expression, ranging between 30 and 90%. All 22 patients were EBV-positive (Fig. 1F) according to ISH detection but negative for Epstein-Barr virus nuclear antigen (EBNA)-2.

### Gene expression profiling

**Figure 2:**
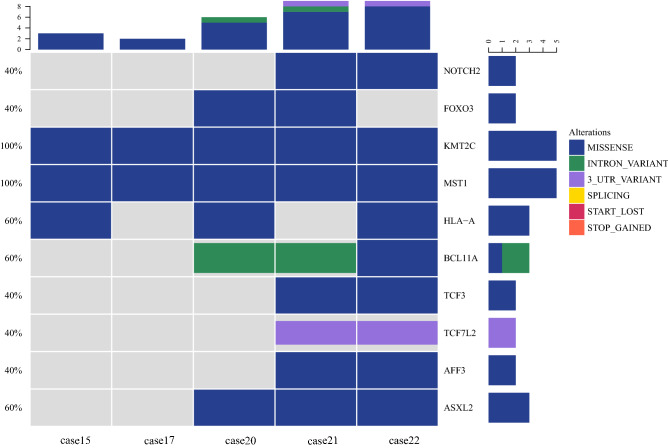
Genes with high mutation frequencies of 5 cases of NKTCL in children and adolescents by NGS.

NGS was performed for 5 cases of NKTCL (Fig. 2). The mutations were arranged based on deleterious SNPs prediction (SIFT < 0.05 and Polyphen2-HDIV > 0.453) and tumor driver genes screening. The results revealed that the high recurrently mutated genes in this cohort were the KMT2C (5/5, 100%; 5 missense), MST1(5/5, 100%; 5 missense), followed by HLA-A (3/5,60%;3 missense), and BCL11A (3/5,60%;1 missense and 2 intron variant).

### Treatment and follow-up

The treatment modalities and the clinical outcomes are summarized in Table 1. In brief, 5 patients underwent combined chemo-radiation therapy, 14 patients received chemotherapy or radiotherapy only, and 3 patients refused to receive any treatment. During the follow-up period ranging from 2 to 67 months, 3 patients lost contact information were excluded from the follow-up study. Seven (36.8%) out of all the 19 patients during the follow-up died of the disease, and the median follow‑up period was 44 months. In univariate analysis, patients treated with radiotherapy/chemotherapy showed significantly inferior OS compared with the untreated patients (*P* = 0.004). In multivariate analysis, region involved, B-symptoms, LDH level, stage, IPI score, and treatment had no prognostic correlation with the outcome (*P* > 0.05).

## Discussion

In line with previous studies, the current study of 22 NKTCL in children and adolescents reported here also demonstrated that the male-to-female ratio was 2.14:1, which indicates that males are more susceptible than females, and the B-symptoms were also observed in most cases (73.3%). All 22 cases exhibited focal symptoms and revealed nasal cavity involvement or non-nasal involvement initially and gradually extended to other sites. Accordingly, 6 cases had the manifestations of non-nasal involvement. It is worthy to note that 3 cases revealed initial symptoms in the lymph nodes area, but subsequent imaging examination showed the spreading of the disease to the nasal sites, which could improve the level of those early diagnoses. The Ann Arbor staging system was used to evaluate the clinical stages of patients: stage I/II was observed in less than 30% of all cases, which is lower than that reported by the previous literature^6–8,11^. It may be presumably related to the restrictive standard of the enrolled group in our study. One of the diagnostic criteria of enrolled patients was multiple site involvement, so most patients in our study had been classified into stage III /IV.

According to the WHO criteria, patients with NKTCL usually exhibit prominent coagulative necrosis and angio-destructive growth pattern in terms of histopathological features. The cytological spectrum is broad with variable-sized cells. In the present study, the most common type of morphological pattern was MC type, which accounted for half of all patients. Most patients (86.4%) showed coagulative necrosis. The current study also revealed that all 22 cases exhibited immunohistochemical features similar to the cases observed in adults, having a positive expression of CD3 and the cytotoxic markers such as granzyme B and TIA-1. Most importantly, the tumor cells were CD56 positive in most cases. CD5 focal positive or negative were expressed in all available cases. It should be noted that 2 patients with NKTCL exhibited strong and diffuse CD30 immunoreactivity. In our previous study, we have reported that the tumor cells were positive for CD30 heterogeneously in most cases of NKTCL, and some cases were CD30 diffuse positive, and this may be confused with anaplastic large cell lymphoma^12^. In the present study, all 22 patients were EBNA-2 negative, as shown by immunohistochemistry analysis. EBV transiently runs a short lytic program and then predominantly establishes latent infection in the primary infection stage, and EBV-infected cells are EBNA-2 positive in type III latency pattern, i.e., infectious mononucleosis (IM) or lymphomas in immunocompromised people^13^. Nevertheless, NKTCL expresses latent membrane protein (LMP)-1 and EBNA-1 with latency II of EBV infection. EBNA-2 is supposed to be negative in this disease^14,15^. Therefore, EBNA-2 may be a useful marker for diagnosis and differential diagnosis of NKTCL.

NKTCL in children and adolescents may be particularly difficult to distinguish from other EBV-related disorders including IM and chronic active EBV infection (CAEBV) because these diseases share similar immunophenotypic markers like CD3, granzyme‑B, TIA‑1, and EBV. Zhou et al. reported 9 patients in China with EBV^+^ T cell lymphoid hyperplasia (TLH) in the upper aerodigestive tract, whose clinical symptoms were similar to those of IM, and all cases were initially misdiagnosed or misjudged as NKTCL^16^. The clinical symptoms may help to distinguish between NKTCL and other EBV-related disorders, including IM, EBV-TLH or CAEBV^17,18^. Besides, the current study demonstrated that patients with NKTCL tend to exhibit coagulative necrosis and reduced expression of CD5, which is very rare in other EBV-related disorders. Due to difficulty in evaluating clinical course of some cases in the early stages of this disease, early diagnosis has become a challenge to pathologists. Case 21 was initially misdiagnosed as EBV acute infection, but subsequent imaging studies demonstrated the involvement of multiple organs with symptoms of hemophagocytic syndrome. Later, the patient was eventually diagnosed as NKTCL. This disease should be diagnosed with sufficient clinical and pathological evidence, or else follow-up observation should be considered. Furthermore, CD30^+^ anaplastic large cell lymphoma should also be excluded as NKTCL as it could also exhibit heterogeneous expression of CD30. ISH for EBV may help to distinguish between the two diseases.

According to NGS, genes with high mutation frequencies in 5 cases of NKTCL were detected. These genes were KMT2C, MST1, HLA-A and BCL11A which are involved in epigenetic modifications, tumor suppression and immune surveillance^19–22^. However, inconsistent with the results of previous studies of NKTCL in adults^23–25^, no mutations in DDX3X and JAK-STAT pathway molecules, which are considered as recurrent mutations in NKTCL, were found in our study. The analysis of molecular signature genes based on the 5 cases may provide a deep understanding of this rare disease. Whether NKTCL in children and adolescents represents a specific type of NKTCL that has characteristic molecular features, requires further study on a larger sample size.

Although some unfavourable prognostic factors, including high LDH level and non-nasal involvement at an advanced stage are reported in NKTCL of adults, and similar studies have not been analyzed well in young patients for limited samples^26–29^. In the present study, 3 out of 7 cases which refused chemotherapy or radiotherapy died of this disease. Moreover, univariate analysis showed that cases without treatment were associated with a more unsatisfactory outcome, suggesting that active treatment is necessary after confirmed diagnoses of children and adolescents with NKTCL. However, no statistically significant association was found between clinical parameters and prognosis; therefore, further investigation with a large sample size is needed.

In conclusion, this study, to our knowledge, displays the clinicopathological and genetic features of this rare disease for the first time. The current study revealed that NKTCL in children and adolescents could involve non-nasal sites and exhibited histopathological and immunohistochemical features similar to the cases in adults. This disease is difficult to distinguish from EBV-related disorders in young people, including IM and CAEBV. Furthermore, active treatment is necessary after the diagnosis of NKTCL is confirmed.

## Supplementary Information

Supplementary Information.

## Data Availability

All data generated or analysed during this study are included in this published article.
